# Utilization of durian seed for *Monascus* fermentation and its application as a functional ingredient in yogurt

**DOI:** 10.1186/s40643-022-00619-y

**Published:** 2022-12-14

**Authors:** Ignatius Srianta, Indah Kuswardani, Susana Ristiarini, Netty Kusumawati, Laura Godelive, Ira Nugerahani

**Affiliations:** Department of Food Technology, Faculty of Agricultural Technology, Widya Mandala Catholic University Surabaya, Jalan Dinoyo 42-44, Surabaya, 60295 Indonesia

**Keywords:** *Monascus*-fermented durian seed, Yogurt, Antioxidant, Phenolic content, *Monascus purpureus*, Refrigerated storage

## Abstract

As a widely consumed fermented milk product, yogurt undergoes constant development to increase its functional properties. *Monascus purpureus*-fermented durian seed, which has been proven to possess antioxidative properties, has the potential to improve yogurt properties. This study aimed to analyze the use of *Monascus*-fermented durian seed (MFDS) as a functional ingredient in yogurt and its effect on physicochemical properties, lactic acid bacteria (LAB) count, antioxidative properties, and consumer acceptability of set-type yogurt during refrigeration. Changes in physicochemical properties, including color, pH, titratable acidity, syneresis, LAB count, total phenolic content (TPC), and antioxidant activity were evaluated at 7-day intervals during 14 days of refrigerated storage (4 °C). Sensory evaluations were carried out for freshly made samples after 7 days of storage. The results showed that the addition of MFDS to yogurt gave significant effects on some of the parameters measured. Yogurt with added MFDS powder produced a more red color (*L* = 88.55 ± 1.28, a* = 2.63 ± 0.17, b* = 11.45 ± 1.15, *c* = 11.75 ± 1.15, *H* = 77.00 ± 0.64), reached the highest TPC (2.21 ± 0.46 mg/GAE g), antioxidant activity (0.0125 ± 0.0032 mg GAE/g), and syneresis (5.24 ± 0.51%) throughout 14 days of storage. The addition of MFDS only gave a slight difference to pH and titratable acidity, while no significant difference was made for LAB count. For sensory evaluation, the addition of MFDS, particularly the ethanol extract, to yogurt was well-liked by panelists. Citrinin content in MFDS yogurt can be decreased under the limits set. Overall, the addition of MFDS has a high potential of improving yogurt properties, particularly its antioxidative properties.

**Graphical Abstract:**

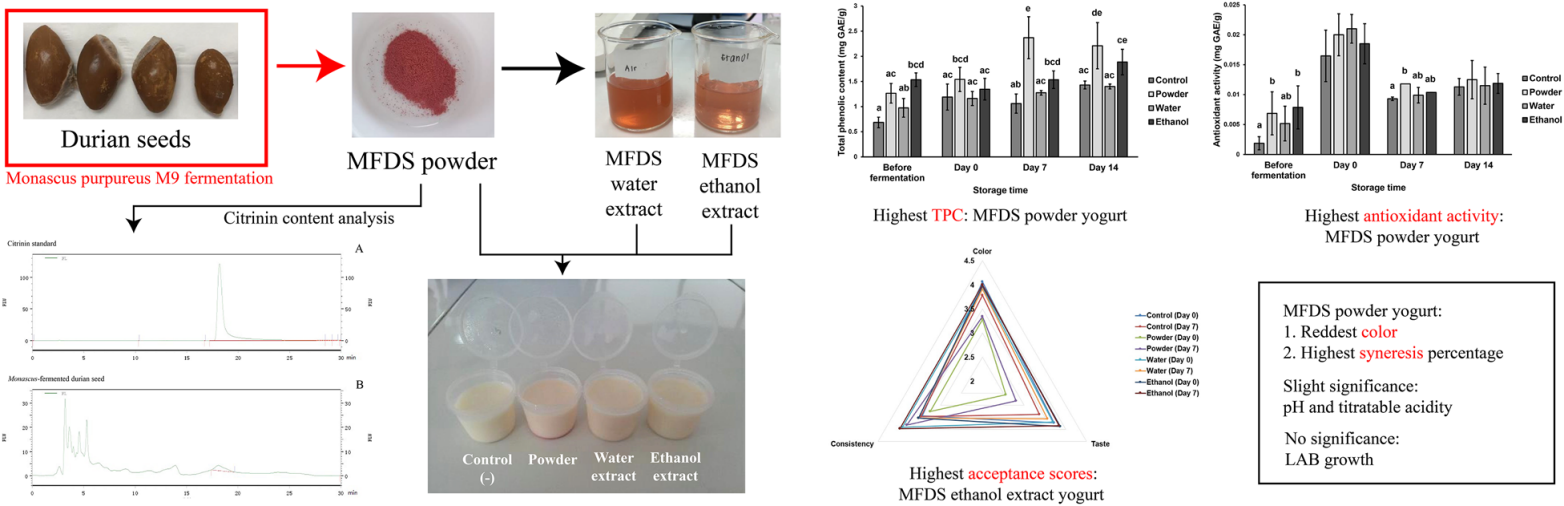

## Introduction

Yogurt is a fermented dairy product usually produced with *Streptococcus thermophilus* and *Lactobacillus delbrueckii subsp. bulgaricus*. Yogurt is rich in activity probiotics and nutritional compounds, such as calcium, protein, and potassium (Gahruie et al. 2015; Liu and Lv 2018). Lactic acid bacteria (LAB) in yogurt are credited with improving digestion, harmful bacteria growth suppression in the gastrointestinal tract, the bioavailability of milk constituents, alleviation of lactose intolerance, cancer suppression, and hypocholesterolemic effects (Shiby and Mishra 2013; Aryana and Olson 2017). Owing to its functional properties, yogurt has been widely consumed for thousands of years and undergoes constant development to improve its health benefits. One of the most common ways to improve yogurt quality is by adding functional ingredients, such as essential oils (Comunian et al. 2017), fruits, flowers (Chouchouli et al. 2013; Marchiani et al. 2015; Sah et al. 2016; Liu and Lv 2018; Dimitrellou et al. 2020), green tea and coffee powders (Dönmez et al. 2017), and *Monascus*-fermented products (Jeon et al. 2011; Abdel-Raheam et al. 2019).

*Monascus*-fermented products are substrates fermented by *Monascus* sp., typically from the three species: *Monascus purpureus*, *Monascus pilosus*, and *Monascus ruber*. The most notable *Monascus* product is red mold rice or commonly known in Indonesia as *angkak*, which is a *Monascus*-fermented rice product. These products have been commonly used as food colorants and dietary material due to the secondary metabolites produced during fermentation: *Monascus* pigments, monacolin K, γ-aminobutyric acid (GABA), and dimerumic acid (Lee and Pan 2012). These compounds have been reported to have bioactivities, such as anti-inflammatory, anticancer, antimicrobial, antiobesity, antidiabetic, and antihypercholesterolemia (Srianta et al. 2021). Jeon et al. (2011) reported on the addition of *Monascus* sp. and *Lactobacillus* sp. fermented Chinese yam (*Dioscorea batatas*) powder in yogurt and found that the addition increased total phenolic content, 2,2-diphenyl-1-picrylhydrazyl (DPPH) scavenging activity, reducing power, angiotensin-converting enzyme (ACE) inhibitory activity, and GABA content of yogurt in comparison to the control. Abdel-Raheam et al. (2019) also reported that the addition of red, orange and yellow pigments from *Monascus ruber* fermentation in yogurt received high acceptability scores on texture, odor, color, taste, and overall acceptability. These results show the potential of *Monascus*-fermented products to be added as a yogurt ingredient, especially with substrates other than rice.

Durian is one of the many tropical fruits largely produced in Indonesia. Waste from durian production can go as high as 70% which consists of shells and seeds (Purnomo et al. 2016). Srianta et al. (2012), Subianto et al. (2013), and Nugerahani et al. (2017) reported that *Monascus purpureus* fermentation on durian seeds is possible and has been proven to exhibit antioxidant, antihypercholesterol, and antidiabetic activities due to *Monascus* pigments, phenolics, and monacolin K. These compounds have different polarities, which could affect MFDS bioactivity according to the extraction solvent (Subianto et al. 2013). This raises the potential of *Monascus*-fermented durian seeds (MFDS) of various forms to be sustainable and functional ingredients for food products, especially yogurts. This study aimed to analyze the use of MFDS as a functional ingredient in yogurt and its effect on physicochemical properties, LAB count, antioxidative properties, and consumer acceptability of set-type yogurt during refrigeration.

## Materials and methods

### *Monascus*-fermented durian seed preparation

The durian seeds of the *Petruk* variety were provided by a local durian product seller in Surabaya, Indonesia. The seeds were washed clean, sorted, and kept in the freezer (Sharp, Japan) at − 20 °C before use. The seeds were thawed for 30 min (30 °C), washed, weighed, and heated with 5% (w/v) Ca(OH)_2_ solution (1:1) for 10 min at 85–90 °C. The seeds were washed and separated from their skin, diced into smaller pieces (1 cm^3^), weighed (50 g/batch) inside 250 mL titration flasks, sterilized with an autoclave (121 °C, 15 lbs/inch^2^, 15 min, Hirayama, Saitama, Japan), and cooled to 30 °C. The sterilized durian seeds were inoculated with 5% (v/w) *Monascus purpureus* M9 (NCBI Accession Number: HM188425.1) and left to ferment for 14 days at 30 °C to obtain MFDS. The MFDS was then dried in an oven for 24 h at 45 °C (Binder, Germany), ground, and sieved (80 mesh) to obtain MFDS powder. MFDS powder would then be analyzed for citrinin content.

MFDS powder was then extracted with two different solvents, water and ethanol. MFDS that had been weighed were extracted with sterile distilled water (1:50 w/v) using a shaking water bath (LabTech, Hopkinton, Massachusetts) at 100 rpm, 40 °C, for 1 h and strained using a vacuum pump to obtain the MFDS water extract. The MFDS ethanol extract was made by extracting MFDS powder with ethanol (1:50 w/v) using the Soxhlet method for 2.5 h. The extract was then evaporated with a rotary evaporator (70 °C, 50 rpm, Buchi, Switzerland) and rehydrated with sterile distilled water. Both MFDS extracts were pasteurized for 30 min at 70 °C in a water bath (Faithful, Hebei, China) and stored at 4 °C.

### Yogurt preparation

Commercial ultra-high temperature sterilized full cream milk (Ultrajaya, Indonesia) was purchased from a local market. Granulated sugar (10% w/v) and skimmed milk powder (2.2% w/v) were added to the milk and mixed thoroughly. The mixture was pasteurized at 90 °C for 5 min above a Bunsen burner fire. Gelatin (0.8% w/v) and MFDS treatments as illustrated in Table 1 were added to the mixture at 80 °C. After it was homogenously mixed, the mixture was taken off the fire and cooled in an ice bath to 41 °C. Commercial yogurt starter (a mixture of *Streptococcus thermophilus*, *Lactobacillus bulgaricus*, and *Lactobacillus acidophilus*, Lallemand, France) of 0.3% (w/v) was inoculated and mixed into the yogurt mixture. The mixture was poured into sterile plastic cups with some put aside for pH analysis. Fermentation was conducted at 42 °C for 4 h to obtain MFDS yogurt. Color, syneresis, titratable acidity, pH, total LAB count, antioxidant activity, phenolic content, and sensory evaluation were determined after fermentation. The yogurt samples were cooled at 4 °C for 14 days and underwent analysis of the parameters mentioned at 7-day intervals.

**Table 1:** *Monascus*-fermented durian seed yogurt formulation

Ingredient	Treatment			
	Control	Powder	Water	Ethanol
Full cream milk (mL)	700			
Granulated sugar (w/v)^a^	10%			
Skimmed milk powder (w/v)	2.2%			
Gelatin (w/v)^a^	0.8%			
MFDS powder (w/v)^b^	0	0.15%	0	0
MFDS water extract (v/v)^b^	0	0	7.5%	0
MFDS ethanol extract (v/v)^b^	0	0	0	7.5%
Water (v/v)^b^	7.5%	6.35%	0	0
Starter culture (w/v)^c^	0.3%			

^a^Calculated based on the milk volume

^b^Calculated based on the milk, sugar, skimmed milk powder, and gelatin total volume

^c^Calculated based on the total volume of the mixture after MFDS treatment

### Physicochemical analysis

Color parameters of lightness (L*), redness (a*), and yellowness (b*) were determined by using a color reader (Konica Minolta, Japan). Chroma and hue parameters were further derived from a* and b* values based on Eqs. (1) and (2):

1 $${\text{Chroma}} = \sqrt {a^{*2} + b^{*2} }$$

2 $${\text{Hue}} = {\text{arctan}}\left( {b^* /a^* } \right)$$

Syneresis analysis was conducted according to Amatayakul et al. (2006). The yogurt samples inside the plastic cups were weighed to obtain the initial weights before the cups were tilted to 45° for whey extraction with a pipette and filtration paper. After extraction, the yogurt samples were weighed to obtain the final weight. Syneresis percentage was calculated according to Eq. (3):

3 $$\% {\text{Syneresis}} = \left\{ {\left( {{\text{initial weight}} - {\text{final weight}}} \right)/\left( {{\text{initial weight}} - {\text{empty cup weight}}} \right)} \right\} \times 100\%$$

The pH of the samples was determined by using a pH meter (Xylem Analytics, Germany). The titratable acidity (lactic acid) was determined according to the methodology suggested by Widagdha and Nisa (2015). Standardized NaOH was used to titrate 10 mL of the sample (previously diluted tenfold) with added phenolphthalein indicator until the solution reaches a stable pink color. The results were expressed as total lactic acid percentages.

### Microbiological analysis

The counts of LAB were done in duplicate using de Man, Rogosa and Sharpe agar and incubated for 48 h at 37 °C. The results were expressed as colony-forming units per milliliter of yogurt (CFU mL^−1^).

### Antioxidant activity

Determination of the samples’ antioxidant activity was done with the DPPH assay according to Subianto et al. (2013). In preparation for the assay, distilled water was added to the samples (1:1), centrifuged for 30 min at 5000 rpm, and filtered with Whatman number 42 filter paper. The supernatants were diluted fivefold to obtain the samples for DPPH assay. The samples and the DPPH solution reacted for 30 min and the absorbance (A) was measured at 517 nm in a UV–Vis spectrophotometer (Shimadzu, Japan). The Gallic acid standard curve was conducted with methanol as the solvent. The samples’ antioxidant activity was expressed in milligrams of Gallic acid equivalent per gram of yogurt (mg GAE·g^−1^) and calculated according to Eq. (4):

4 $$\% {\text{Inhibition}} = \left\{ {\left( {{\text{A control}} - {\text{A sample}}} \right)/\left( {\text{A control}} \right)} \right\} \times 100\% .$$

### Phenolic content

The preparation of the samples was carried out using the same procedure as the samples for the DPPH assay. The samples (0.1 mL) were reacted to 0.5 mL Folin Ciocalteu reagent inside a 10 mL volumetric flask, homogenized, then 1.5 mL of 20% Na_2_CO_3_ and distilled water were added. After homogenization, the solution was put aside for 30 min at room temperature and the absorbance was measured at 750 nm. The Gallic acid standard curve was conducted with distilled water as the solvent. The results were expressed in milligrams of Gallic acid equivalent per gram of yogurt (mg GAE·g^−1^).

### Sensory evaluation

Fifty untrained students of Widya Mandala Surabaya Catholic University who have basic sensory knowledge of yogurt products were instructed to evaluate the color, taste, and consistency of yogurt samples. A hedonic 5-point scale was used, in which ‘5’ represents ‘most like’, ‘4’ represents ‘like’, ‘3’ represents ‘neutral’, ‘2’ represents ‘dislike’, and ‘1’ represents ‘most dislike’ (Setyaningsih et al. 2010).

### Citrinin content

Citrinin content analysis of MFDS was carried out according to Li et al. (2003). MFDS (0.5 g) was extracted with 20 mL of toluene−ethyl acetate−formic acid reagent (7:3:1, v/v/v) and disintegrated for 10 min in an ultrasonic disintegrator. The extract and its container were weighed pre and post-sonication and added with the complex extract reagent to make up for the deficiency. The residue was extracted twice with 15 mL of the reagent and was combined with the initial extract to be centrifuged at 3000 rpm for 20 min. The upper layer of the extract was evaporated and the residue was dissolved in 30 mL of methanol for citrinin determination by high-performance liquid chromatography (HPLC) after filtration.

The HPLC system (Hewlett-Packard 1100, USA) was used for citrinin determination. A reversed phase column (Eclipse XDB C_18_, 4.6 mm × 250 mm, 5 μm) was used after being thermostated at 28 °C in a column oven. The system included a fluorescence detector with an excitation wavelength of 331 nm/500 nm. The samples were eluted with acetonitrile-acidified water (pH 2.5, 35:65, v/v) at a flow rate of 1 mL/min. The detection limit was 50 μg/L.

Citrinin confirmation was done with a liquid chromatography−mass spectrometry (LC–MS) system (Waters ZMD 4000), with an inlet temperature of 120 °C and a dissolvent temperature of 25 °C. The capillary voltage was 3.78 kV and the molecular weights were 100 to 500 Da. The same mobile phase, column, and employed flow rate were used.

### Statistical analysis

The data were analyzed using IBM SPSS Statistics (version 19.0). The added MFDS and storage time of the yogurt samples were the two factors that could affect the parameters tested. Two-way ANOVA was conducted and continued with post hoc pairwise comparisons using Tukey’s HSD test (*p* < 0.05) when the effects were significant.

## Results and discussion

### Color analysis

For all food products, including yogurt, color is one of the visual attributes which has a part in influencing consumers’ preferences. Data of each yogurt sample’s L* (lightness), a* (redness), b* (yellowness), c (chroma), and H (hue) are shown in Table 2. Treatments of MFDS forms had a significant difference in the L*, a*, b*, and h values of yogurt, particularly in the a* value. MFDS has a bright red color from the pigments produced by *Monascus purpureus* during fermentation e.g. the yellow pigments: monascin and ankaflavin, orange pigments: rubropunctatin and monascorubrin, and the red pigments: rubropunctamine and monascorubramine (Feng et al. 2012). These added pigments would give a significant effect on the yogurt color in comparison to the control. Yogurt with added MFDS powder also produced the highest a* values and the lowest H values throughout storage, while yogurt with added MFDS water extract produced the second highest a* values. Overall, yogurt with added MFDS powder produced a redder color compared to other yogurt samples.

**Table 2:** Changes in lightness (*L*), redness (a*), yellowness (b*), chroma (*c*), and hue (*H*) of yogurt samples during storage (4 °C)

Color parameter	Storage time (day)	Treatment			
		Control	Powder	Water	Ethanol
*L*	Before fermentation	89.32 ± 0.93^abcd^	88.12 ± 0.53^a^	88.10 ± 2.14^a^	89.60 ± 1.11^abcd^
	0	89.32 ± 1.63^abcd^	89.45 ± 0.93^abcd^	90.63 ± 0.38^cde^	89.93 ± 1.94^bcde^
	7	90.68 ± 0.77^de^	88.65 ± 0.89^ab^	89.03 ± 1.53^abc^	91.35 ± 0.94^e^
	14	90.00 ± 1.18^bcde^	88.55 ± 1.28^ab^	90.40 ± 0.57^cde^	90.88 ± 0.53^de^
a*	Before fermentation	− 0.33 ± 0.54^a^	2.38 ± 0.10^fg^	1.25 ± 0.16^e^	-0.03 ± 0.22^ab^
	0	0.17 ± 0.23^abc^	2.08 ± 0.34^f^	1.30 ± 0.70^e^	0.18 ± 0.12^abc^
	7	0.73 ± 0.12^d^	2.25 ± 0.05^fg^	1.52 ± 0.17^e^	0.53 ± 0.26^cd^
	14	0.12 ± 0.24^abc^	2.63 ± 0.17^g^	1.33 ± 0.26^e^	0.38 ± 0.50^bcd^
b*	Before fermentation	11.13 ± 0.62^bA^	9.68 ± 0.39^aA^	10.22 ± 0.59^abA^	10.70 ± 0.98^ab^
	0	11.98 ± 0.39^bB^	10.90 ± 0.74^aB^	10.90 ± 1.27^aAB^	11.18 ± 0.93^a^
	7	12.08 ± 0.39^B^	11.62 ± 0.82^B^	11.82 ± 0.63^B^	11.45 ± 0.89
	14	11.58 ± 0.15^AB^	11.45 ± 1.15^B^	11.53 ± 0.90^AB^	11.43 ± 0.72
*C*	Before fermentation	11.15 ± 0.60^A^	9.98 ± 0.39^A^	10.32 ± 0.59	10.70 ± 0.98
	0	11.98 ± 0.39^BC^	11.87 ± 1.82^B^	11.30 ± 1.84	11.25 ± 1.02
	7	12.08 ± 0.39^C^	11.82 ± 0.82^B^	11.92 ± 0.63	11.45 ± 0.89
	14	11.58 ± 0.15^AB^	11.75 ± 1.15^AB^	11.63 ± 0.90	11.43 ± 0.72
*H*	Before fermentation	87.48 ± 2.27^de^	76.18 ± 0.18^a^	83.03 ± 0.90^c^	90.27 ± 1.23^f^
	0	88.75 ± 0.79^def^	78.70 ± 1.86^b^	83.37 ± 3.14^c^	88.88 ± 0.76^ef^
	7	86.52 ± 0.68^d^	78.98 ± 0.87^b^	82.67 ± 1.15^c^	87.42 ± 1.09^de^
	14	89.43 ± 1.18^ef^	77.00 ± 0.64^ab^	83.32 ± 1.74^c^	88.20 ± 2.36^def^

^a–^^f^Mean values with different letters are significantly different (*p* < 0.05)

^A,^ ^B^Mean values in the same column with different letters are significantly different (*p* < 0.05)

The extraction process of MFDS could decrease the color intensity of the MFDS water and ethanol extracts by losing some of the pigments during extraction. Some pigments may have been degraded from the high temperature of extraction and pasteurization. Most *Monascus* pigments are unstable outside of 30–60 °C (Feng et al. 2012). Also, the pigments may have not been fully extracted from the MFDS powder due to the solvent used. Srianta et al. (2019) found that MFDS extracted using ethanol:water solvent with a ratio of 7:3 produced higher red pigment content compared to extraction using a 10:0 ratio solvent. Meanwhile, MFDS powder was added straight into the yogurt mixture, thus most of the pigments were able to be incorporated into the yogurt without any further degradation from the extraction process.

Yogurt with added ethanol extract was less red in comparison to the yogurt with added water extract. The six main *Monascus* pigments are ethanol soluble, but reactions with –COOH or NH_3_ groups of amino acids would produce water-soluble derivative pigments (Feng et al. 2012). Srianta et al. (2012) found more water-soluble yellow, orange, and red pigments in MFDS, which might be due to the amino acid content in durian seeds as a source of NH_3_. A study by Mirhosseini et al. (2013) found that leucine, lysine, aspartic acid, glycine, alanine, glutamic acid, valine, proline, serine, threonine, isoleucine, and phenylalanine were the most abundant amino acids in the chemical structure of durian seed gum. More pigments were extracted with distilled water, resulting in yogurt with a redder color than using the ethanol extract.

### Total phenolic content

**Fig. 1:**
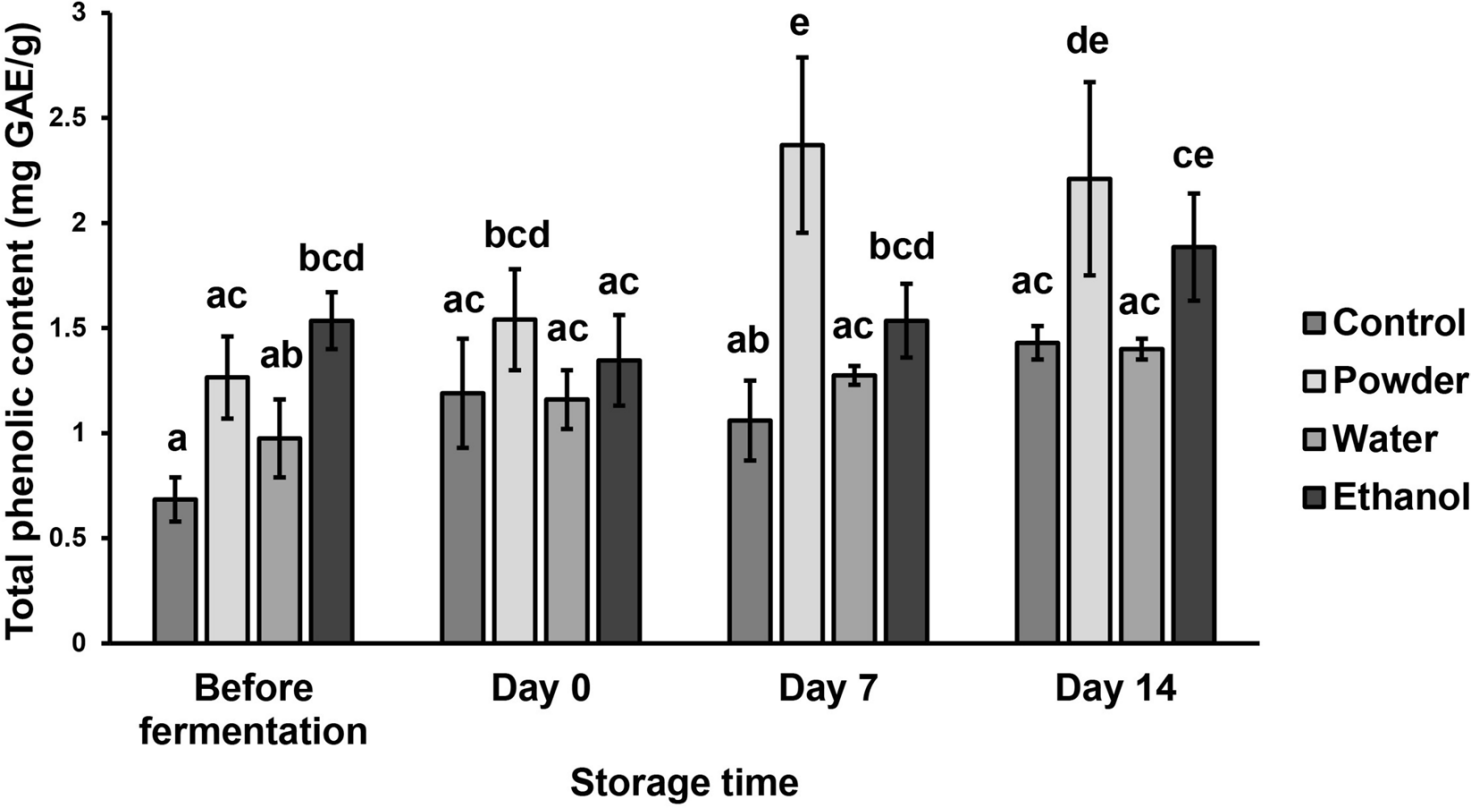
Total phenolic content of yogurt samples during storage (4 °C). Bars with different letters are significantly different (*p* < 0.05)

The total phenolic content data of all yogurt samples is shown in Fig. 1. Yogurt with added MFDS powder reached the highest total phenolic content (TPC) throughout 14 days of storage, with 1.54 ± 0.24 mg GAE/g on day 0, 2.37 ± 0.42 mg GAE/g on day 7, and 2.21 ± 0.46 mg/GAE g on day 14. Yogurt with added MFDS ethanol extract reached the second highest TPC (1.35 ± 0.21, 1.53 ± 0.17, and 1.89 ± 0.25 mg GAE/g on day 0, day 7, and day 14, respectively), while the addition of MFDS water extract did not affect TPC in comparison to the control. MFDS water extract and ethanol extracts were reported to have TPC as high as 3.58 mg GAE/g and 3.61 mg GAE/g, respectively (Srianta et al. 2014).

Durian seeds contain natural phenolics and exhibit antioxidant activity (Subianto et al. 2013). According to Ramli et al. (2021), freeze-dried and rotary evaporated durian seeds had TPC of 4.46 mg GAE/mL and 29.52 mg GAE/mL, respectively. The TPC of durian seeds from previous studies being higher than MFDS extracts might be due to differences in the extraction process and the variety of durian used. Another study by Srianta et al. (2013) compared durian seed water and ethanol extract with MFDS water and ethanol extract. MFDS water extract (589.8 μg GAE/mL) and ethanol extract (473.3 μg GAE/mL) exhibited higher TPC than durian seed water extract (325.8 μg GAE/mL) and ethanol extract (132.0 μg GAE/mL). *Monascus* sp. fermentation can further increase the TPC of substrates and their bioavailability by releasing enzymes including amylases, cellulases, esterases, tannases, and glucosidases to break down cell walls and facilitate phenol extraction (Suraiya et al. 2018; Zhihao et al. 2022). These enzymes can also break bonds between phenolic compounds and other groups such as polysaccharides, lipids, and amines (Cheng et al. 2022; Wang et al. 2018). *Monascus* sp. can also produce micromolecular phenolic compounds by itself (Bei et al. 2017).

The addition of MFDS powder in yogurt resulted in the highest TPC of all the samples, which is due to fewer processing steps of MFDS before being incorporated into the yogurt. A larger amount of phenolic compounds in the MFDS powder could have been preserved and thus able to be detected during the TPC analysis. Ethanol and water extracts of MFDS underwent a few more heat processing steps and would have lost more phenolics due to degradation. Based on the TPC of the yogurt samples, it is observed that phenolics in MFDS were more ethanol soluble rather than water soluble. Abd Razak et al. (2015) also found similar results where methanol extracts of rice bran fermented with *Rhizopus oligosporus* and *Monascus purpureus* had higher amounts of TPC compared to the water extracts.

TPC of yogurt with added MFDS powder and ethanol extract experienced an increase throughout 14 days of storage. The addition of MFDS powder increased the TPC of yogurt from 1.54 ± 0.24 to 2.21 ± 0.46 mg/GAE g during 14 days of storage, while MFDS ethanol extract increased the TPC from 1.35 ± 0.21 to 1.89 ± 0.25 mg GAE/g. Aside from the phenolic compounds present in MFDS, the degradation of milk proteins during fermentation by LAB could release phenolic amino acids and nonphenolic compounds which could have interfered during TPC analysis (Shori 2013; Baba et al. 2014). Similar results were found by Szołtysik et al. (2020), in which the phenolic compounds (anthocyanins, favan-3-ols, ellagitannins, and flavonols) in yogurt with added *Rosa spinosissima* extract experienced a slight elevation after 14 days of storage. Yogurt with added *Azadirachta indica* also experienced an increase in TPC from 38.5 ± 7.2 μg GAE/mL on day 14 to 74.9 ± 6.2 μg GAE/mL by day 28 (Shori and Baba 2013).

### Antioxidant activity

**Fig. 2:**
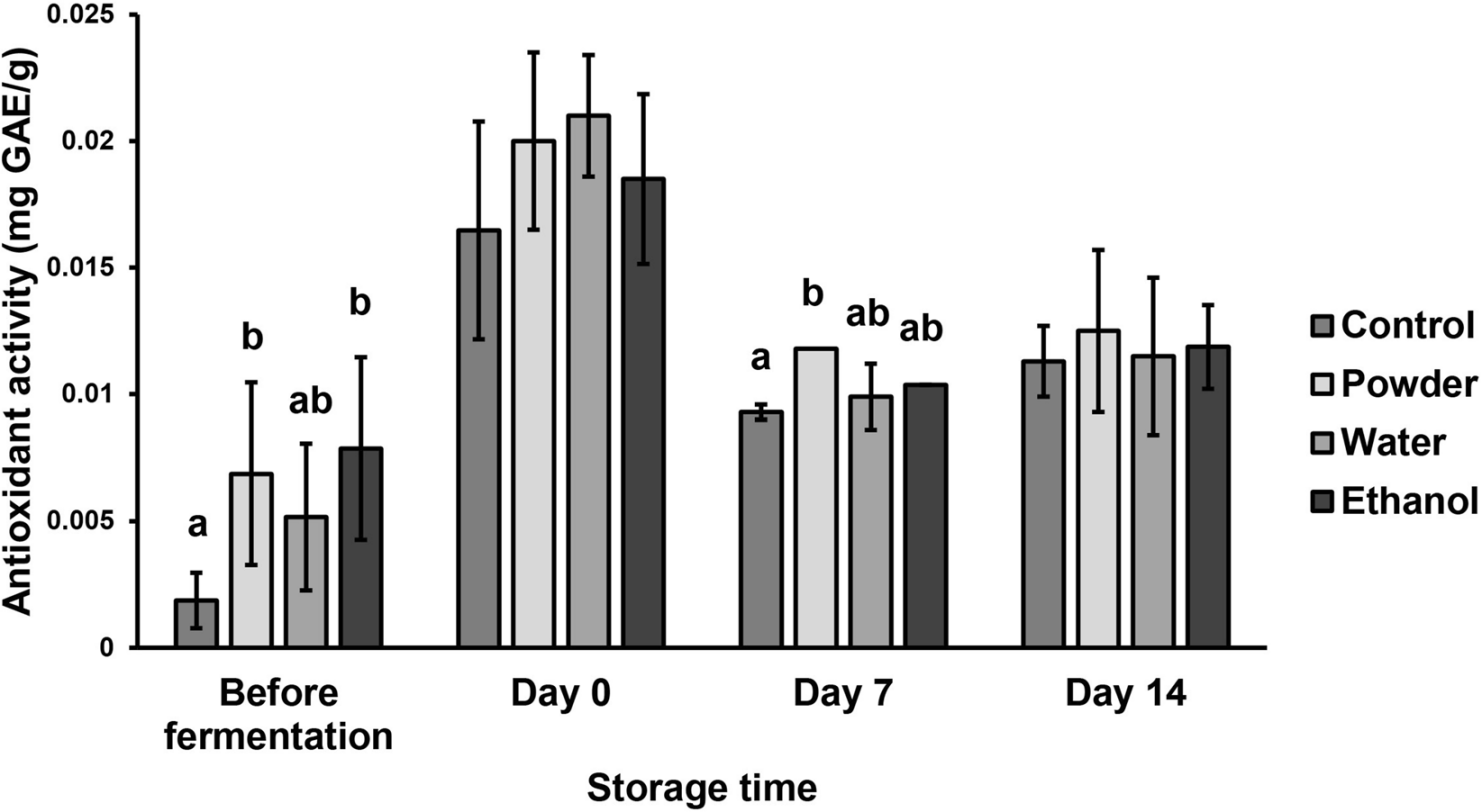
Antioxidant activity of yogurt samples during storage (4 °C). Bars within each storage time with different letters are significantly different (*p* < 0.05)

The antioxidant activity of yogurt samples was determined based on their DPPH scavenging capacities and are shown in Fig. 2. On day 0, yogurt with added MFDS water extract reached the highest antioxidant activity (0.0210 ± 0.0035 mg GAE/g) while the control had the lowest activity (0.0165 ± 0.0043 mg GAE/g). Through day 7 and day 14, yogurt with added MFDS powder produced the highest antioxidant activity (0.0118 and 0.0125 ± 0.0032 mg GAE/g respectively), while the control reached the lowest values with 0.0093 ± 0.0003 mg GAE/g on day 7 and 0.113 ± 0.0014 mg GAE/g on day 14. The addition of any form of MFDS increased the antioxidant activity of yogurt, which was due to the bioactive compounds produced by *M. purpureus* which are *Monascus* pigments, GABA, and monacolins. Srianta et al. (2017) reported that *Monascus purpureus* M9 pigments monapilol B and rubropunctamine were the main antioxidants of *Monascus*-fermented products among the 12 pigments detected (rubropunctatin, monascorubrin, rubropunctamine, monascorubramine, monascin, ankaflavin, xanthomonascin A, xanthomonascin B, yellow II, and monapilol B). The two pigments had a high correlation with the DPPH radical scavenging activity. Tan et al. (2018) found that water-soluble yellow *Monascus* pigments exhibited DPPH and 2,2′-azino-bis(3-ethylbenzothiazoline-6-sulfonic acid) (ABTS^+^) radical scavenging activity, reaching up to 86.33% inhibition from 1 mg/mL sample and 99.95% from 0.25 mg/mL respectively. Other secondary metabolites such as GABA, monacolin K, and dihydromonacolin MV also contribute to DPPH radical scavenging (Srianta et al. 2014). Phenolics from *Monascus*-fermented oats were also reported to have a high correlation with DPPH and ABTS^+^ radical scavenging activity (Bei et al. 2017).

After fermentation, the antioxidant activities of all the yogurt samples were significantly higher than the initial yogurt mixture. The increase in antioxidant activity of the yogurt mixture after fermentation was due to LAB activity. Using the thiobarbituric acid method, Kim et al. (2005) found that *Lactobacillus bulgaricus* and *Lactobacillus acidophilus* exhibit antioxidant activity (81.30% and 65.32% respectively). They can demonstrate radical scavenging activity, inhibitory activity towards lipid peroxidation, show strong reducing power, and produce proteolytic enzymes which help release antioxidative milk peptides (Virtanen et al. 2007; Zhang et al. 2011; Aloglu and Oner 2011). Gjorgievski et al. (2014) found that yogurt fermented with *Streptococcus thermophilus* and *L. bulgaricus* mix culture and *L. acidophilus* monoculture also exhibited antioxidant activity by DPPH radical scavenging (52.44% and 63.99% respectively).

The antioxidant activity of the yogurt samples then significantly decreased by day 7 and stayed stagnant between day 7 and day 14. This data has a negative correlation with the TPC of yogurt samples throughout storage time, in which yogurt with added MFDS powder and ethanol extract had increasing TPC up until day 14. This suggests that the phenolics detected in yogurt with added MFDS did not have DPPH radical scavenging properties. Bei et al. (2017) found that the bound phenolic fraction of fermented oats with *Monascus anka*, mainly ferulic acid, showed greater ABTS^+^ scavenging activity than DPPH radical scavenging activity. Abd Razak et al. (2015) reported that TPC and radical-scavenging activity of rice bran fermented with *R. oligosporus* and *M. purpureus* were poorly correlated for the water and methanol extracts, which might be due to many factors such as the concentration and chemical structures of the phenolics detected. This result suggests that other antioxidant compounds in MFDS might have stronger DPPH scavenging activity than the phenolics detected i.e. pigments, monacolins, and GABA (Srianta et al. 2014; Suraiya et al. 2018). These compounds would have been degraded during storage, thereby decreasing the antioxidant activity of yogurt samples. Similar results have been observed by Chouchouli et al. (2013), in which the antiradical capacities of grape seed-fortified full-fat yogurt decreased during 32 days of storage time (from 1487.4 ± 38.2–1567 ± 52.8 mg TE/100 g on day 1 to 234.8 ± 20.6–440.5 ± 40.3 mg TE/100 g by day 32).

### pH and titratable acidity

**Fig. 3:**
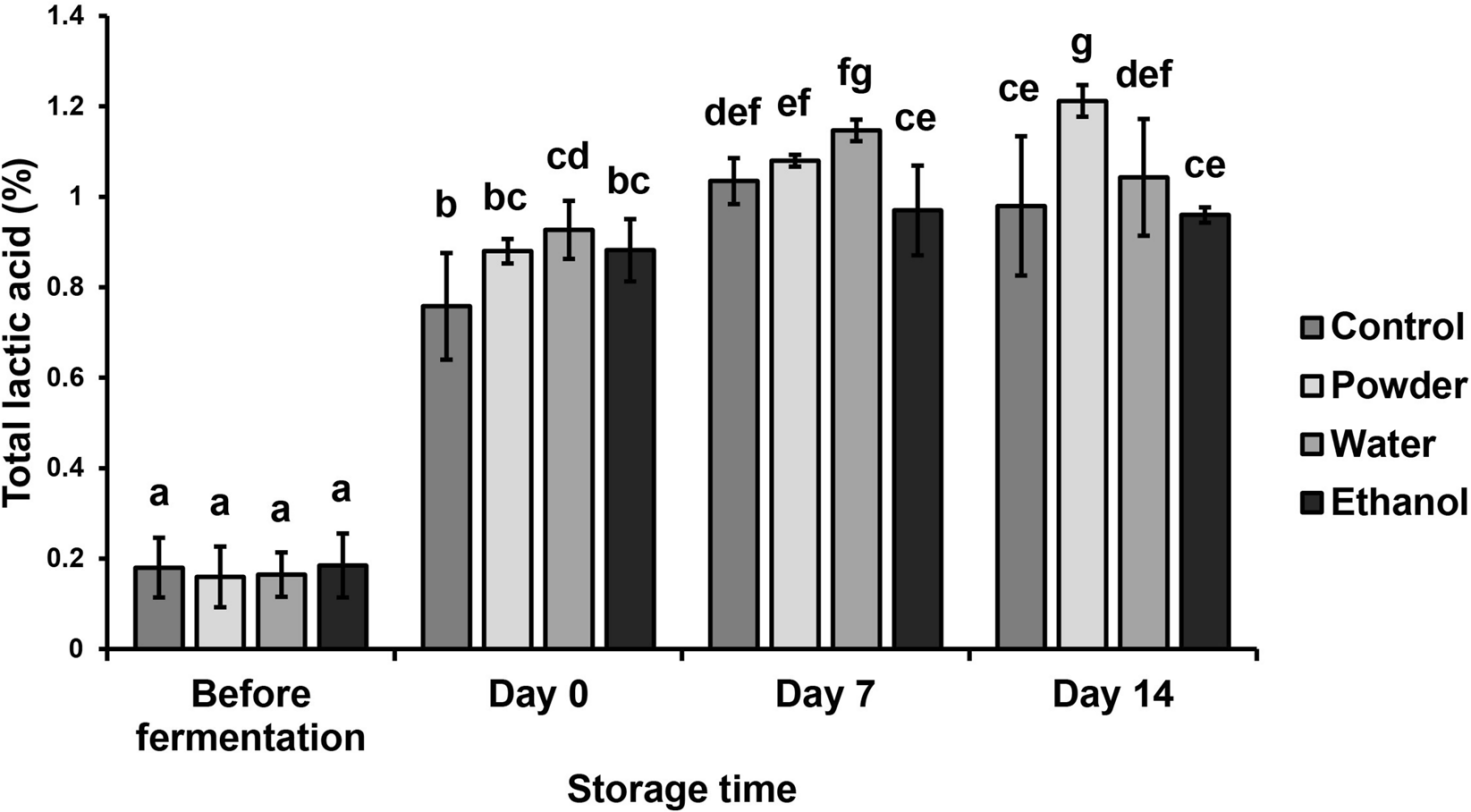
Titratable acidity of yogurt samples during storage (4 °C). Bars with different letters are significantly different (*p* < 0.05)

Changes in yogurt pH added with different MFDS forms are shown in Table 3. pH of the unfermented yogurt mixture decreased significantly after fermentation due to the formation of acids by LAB. pH data from yogurt with added MFDS powder experienced the biggest reduction during storage time from 4.492 ± 0.093 on day 0 to 4.175 ± 0.087 by day 14. Yogurt with added water extract reached the lowest pH on day 0 and day 7, while MFDS powder yogurt has the lowest pH by day 14. These results were in line with the titratable acidity data shown in Fig. 3. The majority of acids found in yogurt are lactic acids, produced by LAB by converting lactose in milk (Chen et al. 2017). The total lactic acid percentage of all yogurt mixture samples increased significantly after fermentation and increased gradually throughout storage time, which is in line with the pH data. The highest total acid percentage was reached by MFDS powder yogurt by day 14. Overall, the addition of MFDS forms to yogurt only gave a slight effect on the pH and titratable acidity in comparison to the control. Darwish et al. (2017) and Melani et al. (2021) found that the addition of *angkak* (*Monascus*-fermented rice/red mold rice) did not give a significant effect on the pH value and acidity of yogurt and goat milk kefir respectively. Pyo and Song (2009) found that the addition of *Monascus*-fermented soybean decreased the pH and increased the acidity of yogurt, while Jeon et al. (2011) found that the pH of yogurt increased after the addition of *Monascus*-fermented Chinese yam. Different substrates of *Monascus*-fermentation will produce different amounts and types of compounds which could affect the characteristics of each *Monascus*-fermented product.

**Table 3:** Changes in pH, syneresis percentage, and total plate count of yogurt samples during storage (4 °C)

Parameter	Treatment	Storage time (day)			
		Before fermentation	0	7	14
pH	Control	6.397 ± 0.048^c^	4.468 ± 0.068^bAB^	4.291 ± 0.096^aAB^	4.289 ± 0.130^aAB^
	Powder	6.415 ± 0.034^c^	4.492 ± 0.093^bB^	4.275 ± 0.111^aAB^	4.175 ± 0.087^aA^
	Water	6.396 ± 0.032^c^	4.353 ± 0.028^bA^	4.215 ± 0.045^aA^	4.249 ± 0.097^aAB^
	Ethanol	6.389 ± 0.033^b^	4.473 ± 0.105^aAB^	4.390 ± 0.089^aB^	4.373 ± 0.087^aB^
Syneresis (%)	Control	–	2.22 ± 0.63^A^	2.42 ± 0.60^A^	2.56 ± 0.68^A^
	Powder	–	3.29 ± 0.43^aB^	3.10 ± 0.27^aA^	5.24 ± 0.51^bB^
	Water	–	2.83 ± 0.82^AB^	2.67 ± 0.50^A^	3.60 ± 0.60^AB^
	Ethanol	–	2.59 ± 0.46^A^	2.68 ± 0.60^A^	2.92 ± 0.69^A^
Total plate count (CFU/mL)	Control	2.65 × 10^7a^	6.27 × 10^9b^	3.36 × 10^10bc^	1.96 × 10^11c^
	Powder	3.05 × 10^6a^	6.22 × 10^9a^	2.04 × 10^10a^	1.90 × 10^7a^
	Water	1.34 × 10^6a^	7.82 × 10^10b^	4.84 × 10^10b^	1.16 × 10^9ab^
	Ethanol	4.00 × 10^6a^	1.55 × 10^10bc^	1.12 × 10^11c^	1.22 × 10^9b^

^a–^^c^Mean values in the same row with different letters are significantly different (*p* < 0.05)

^A,^ ^B^Mean values in the same column with different letters are significantly different (*p* < 0.05)

### Syneresis

As a commercial product, syneresis percentage is best kept at a minimum for yogurt to suit consumer preferences. Table 3 shows the effect of different MFDS forms addition to syneresis percentage in yogurt. The addition of MFDS forms gave a significant effect on the syneresis percentage of yogurt samples, although only yogurt with added MFDS powder experienced an increase in syneresis percentage by day 14. Syneresis percentages of yogurt with added MFDS powder (3.29 ± 0.43–5.24 ± 0.51%) throughout storage are higher compared to other yogurt samples. Syneresis takes place due to the weakening of the yogurt gel structure thus losing the ability to entrap water (Sah et al. 2016). A higher syneresis percentage found in yogurt with added MFDS powder might be related to its TPC. Yogurt with MFDS powder reached the highest TPC out of all the samples (1.54 ± 0.24–2.21 ± 0.46 mg/GAE g) and a high amount of phenolic compounds has been proven to affect yogurt syneresis by other studies. Increased rates of syneresis were detected in yogurts with added ingredients containing phenolic compounds such as blueberry juice, green tea powder, *Ferulago angulata* extract, and grape pomace (Marchiani et al. 2015; Jeong et al. 2018; Dimitrellou et al. 2020; Keshavarzi et al. 2020). Excess polyphenol concentrations could reduce the gel matrix that confines the yogurt serum by decreasing the volume of each protein–phenolic cage and preventing gel matrix formation (Jeong et al. 2018). Increasing the number of particle–particle junctions in the gel structure could lead to the shrinkage of the network and dismissing interstitial liquid (Dönmez et al. 2017).

### Total plate count

Changes in the total plate count of yogurt with added MFDS are shown in Table 3. The addition of MFDS forms to yogurt did not give a significant difference in the total plate count in comparison to the control. The total plate count yogurt mixture increased after fermentation for all yogurt samples. However, the total plate count of yogurt with added MFDS did not increase significantly from day 0 to day 7. By day 14, the total plate count of all samples except the control experienced a decrease. Yogurt with added MFDS powder was not significantly affected during storage time even though it produced the same pattern of data as the other samples. The addition of MFDS powder to yogurt resulted in the lowest total plate count reached, from 6.22 × 10^9^ on day 0 to 1.90 × 10^7^ on day 14). Yogurt without any added MFDS experienced an increase in the total plate count by day 14, reaching 1.96 × 10^11^ from 6.27 × 10^9^ on day 0.

*Monascus* fermentation produces various compounds, among them are pigments and phenolics which have antibacterial characteristics. These compounds may have inhibited the growth of LAB during storage. Pigments are the major bioactive compounds found in *Monascus*-fermented products along with monacolins (Zhu et al. 2019), with each group of pigments having different antibacterial mechanisms. Kim et al. (2006) found that amino acid derivatives of red *Monascus* pigments can suppress the growth of Gram-positive bacteria, which indicates that the red pigment derivatives from MFDS could have inhibited LAB growth. The hydrophobicity of bacteria cell surfaces increases when the pigments are adsorbed into the cell, which leads to cell aggregation into pellets. Pellet formation of cells results in the limited transfer of oxygen and nutrients into bacteria cells. Orange pigments have a good affinity with liposomes, resulting in an interaction with the phospholipid of bacteria cytoplasmic membranes. The interaction disrupts the bacteria membrane and causes cellular leakage. Orange pigments can also stimulate pellet formation in cells (Zhao et al. 2016). On the other hand, phenolics can disrupt cytoplasmic membrane and cause lysis, and interact with enzymes, substrates, and metal ions which prevents bacteria metabolism (Vaquero et al. 2007; Oulahal and Degraeve 2022; Melani et al. 2021).

MFDS water and ethanol extracts underwent further processing, starting with the extraction of compounds with various polarities using purely polar or nonpolar solvents and then being treated to sterilization. These caused the number of compounds in the extracts to be less than in the MFDS powder. This would explain why the addition of MFDS powder suppressed the growth of LAB in yogurt the most. Melani et al. (2021) found that the total plate count of *angkak*-supplemented kefir also decreased during storage time. Pyo and Song (2009), however, found that *Monascus*-fermented soybeans had an increase in viable LAB due to the free and essential amino acids produced from *Monascus* fermentation being a source of nutrients. According to the National Standardization Agency of Indonesia (BSN), the minimal amount of starter bacteria in yogurt is 10^7^ log/g (BSN 2009) thus MFDS-supplemented yogurt is by the standard.

### Sensory evaluation

**Fig. 4:**
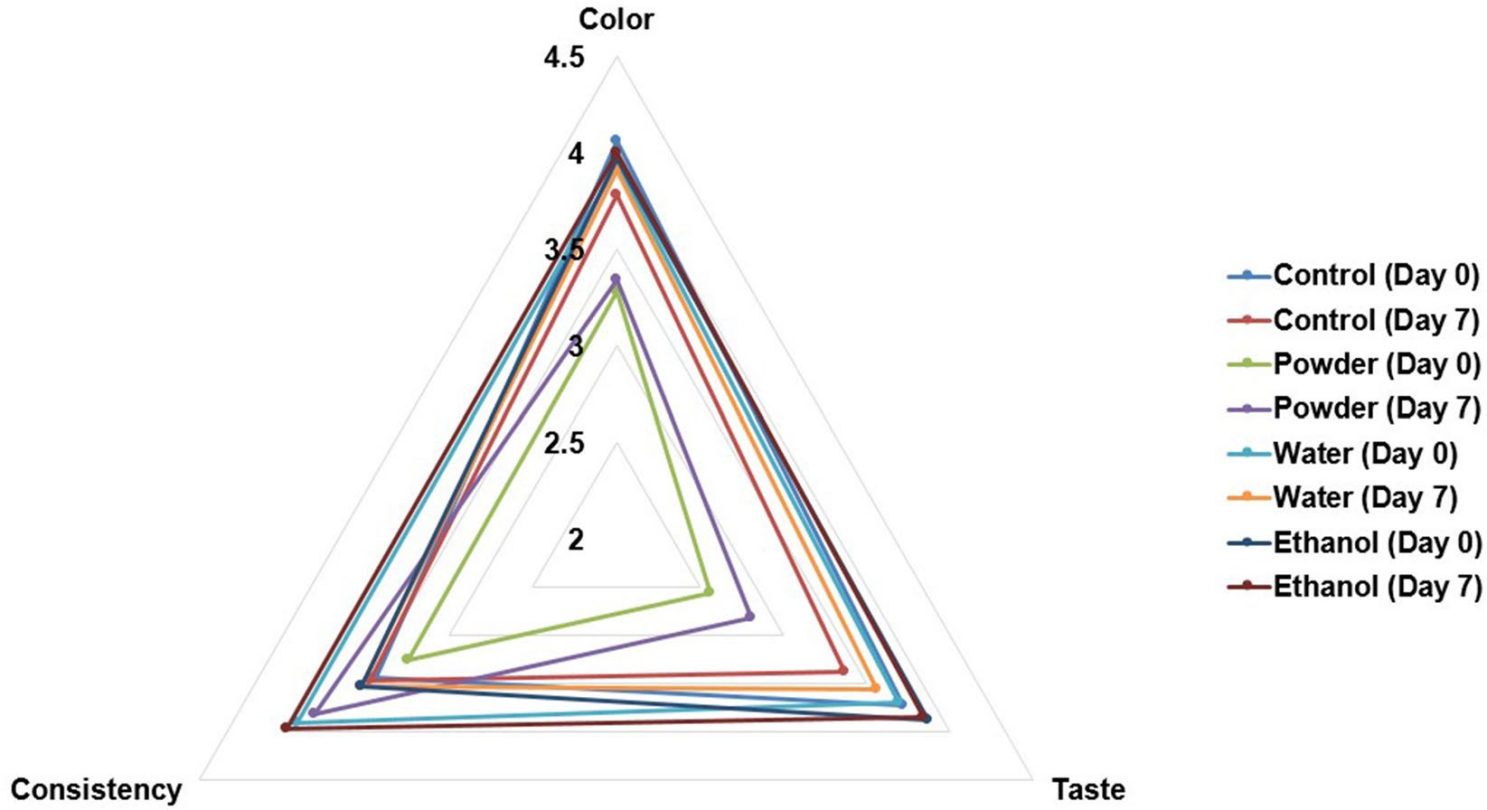
Sensory evaluation data for color, taste, and consistency of yogurt samples during storage (4 °C)

According to Fig. 4, the acceptance scores of all yogurt samples were in the range of 2.56 (dislike-neutral) to 4.06 (like-most like). The addition of MFDS powder had a significant difference in the panelist acceptance score for color and taste, while the addition of any form of MFDS did not give a significant effect on the consistency acceptance score. Storage time did not give a significant effect on the panelist acceptance scores, which meant the quality of all yogurt samples remained stable after seven days of storage. Yogurt with added MFDS powder obtained the lowest acceptance scores for color (3.28–3.34) and taste (2.56–2.81) on days 0 and 7 respectively. The highest score for each parameter was reached by yogurt with added ethanol extract, with scores of 3.97–4.00 for color, 3.87–3.84 for taste, and 3.53–3.97 for consistency. The results show that the addition of MFDS, particularly the ethanol extract, to yogurt was well-liked by panelists.

Color analysis results from Table 2 show that yogurt with added MFDS powder produced a more red-colored yogurt in comparison to the other samples. Without the information about any flavor or added ingredients in yogurt, panelists could have an expectation of a white color regularly found in plain yogurt. A difference in the color of yogurt with added MFDS powder in comparison to other samples might affect panelists’ acceptance scores. MFDS powder was not incorporated evenly in the yogurt, which lead to the accumulation of powder at the bottom of the yogurt cup. This overall appearance might also affect the scores. Panelists have also commented that yogurt with added MFDS powder had a bitter taste. The bitterness might be due to the presence of phenolics in the yogurt (Li and Duan 2018). According to the total phenolic content data from Fig. 2, yogurt with added MFDS powder was found to have the highest TPC out of all the samples. Reginio et al. (2016) also reported that bitterness and astringency were detected by panelists from *Monascus* biopigment beverages.

### Citrinin content of Monascus-fermented durian seed

**Fig. 5:**
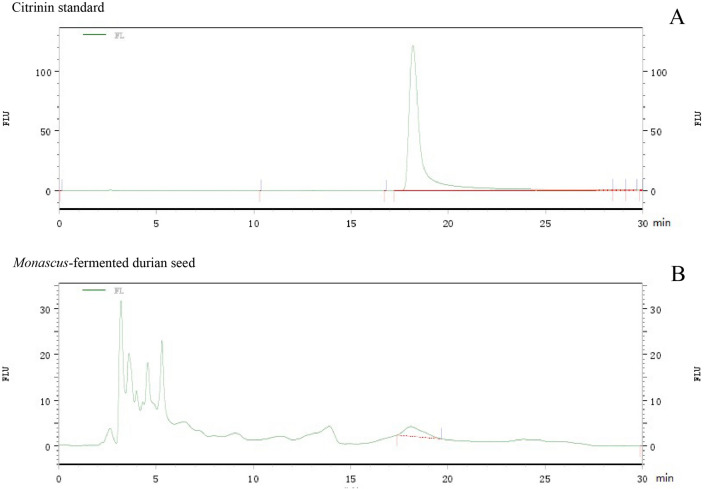
HPLC analysis of citrinin standard (**A**) and citrinin content in *Monascus*-fermented durian seed (**B**)

A notable secondary metabolite from *Monascus purpureus* not yet discussed is citrinin. Citrinin is a mycotoxin produced from the same polyketide biosynthesis pathway as *Monascus* pigments and monacolin K (Agboyibor et al. 2018). Citrinin has cytotoxic, nephrotoxic, hepatotoxic, carcinogenic, and immunosuppression effects when ingested by humans and animals (Magro et al. 2016). It is essential to analyze the citrinin content of MFDS to ensure the safety of MFDS yogurt. HPLC analysis (Fig. 5) shows that the citrinin concentration of MFDS is 5.53 ppm.

Countries have different regulations for citrinin content in *angkak*. The citrinin limit in Taiwan is 2 ppm, 0.2 ppm in Japan, and 0.02 mg/kg body weight per day in Europe (Lee et al. 2010; Pattanagul et al. 2007; European Food Safety Authority 2012). The citrinin concentration of MFDS is higher than the limits mentioned, thus not recommended for high and frequent amounts of consumption. However, the MFDS extraction process with a 1:50 (w/v) ratio of MFDS and solvent produced MFDS yogurt with an estimated 0.1106 ppm of citrinin, which is below the limit in Taiwan and Japan. Citrinin has been stated to be unstable and thermolabile (Doughari 2015; Zhang et al. 2021). According to Trivedi et al. (1993a, b), citrinin heated at 80 °C for 60 min in watery conditions resulted in a 50% reduction but did not affect the citrinin cytotoxicity, while the heating temperatures of 90–110 °C increased the cytotoxicity. Citrinin H1, a highly toxic compound formed from citrinin degradation, was found after heating at 140 °C with the presence of water or at 100 °C for 30 min (Trivedi et al. 1993a, b). Shu and Lin (2002) found that citrinin concentration in *angkak* was dramatically decreased after boiling in water, while Hirota et al. (2002) stated that citrinin degrades on heating above 80 °C under aqueous conditions. The processing heat of 80 °C during yogurt production would neither increase nor decrease the cytotoxicity of MFDS yogurt but might have slightly decreased citrinin content. Further research should be done regarding the amount of citrinin content in MFDS yogurt.

## Conclusion

In this study, the addition of MFDS powder to yogurt produced the largest improvement in yogurt parameters, particularly its antioxidative properties, among the other forms of MFDS. Besides producing a more interesting appearance of yogurt from the red color MFDS imparts, TPC and antioxidant activity were also increased due to less loss of phenolic compounds in MFDS powder in comparison to other forms which undergo the extraction process. Due to higher phenolic content, yogurt with added MFDS powder also experienced the highest syneresis percentage and imparted a bitter taste, which was reflected in the lowest acceptance scores by panelists. MFDS powder had a high citrinin concentration. On the other hand, yogurt with added MFDS ethanol extract had the highest acceptance scores and the extracts had a lower citrinin concentration than the limits set. MFDS has the potential as a functional ingredient for food, especially yogurt.

## Data Availability

The datasets generated during and/or analyzed during the current study are available from the corresponding author upon reasonable request.

## Abbreviations

MFDS: *Monascus*-fermented durian seed
LAB: Lactic acid bacteria
GABA: γ-Aminobutyric acid
DPPH: Diphenyl-1-picrylhydrazyl
ACE: Angiotensin-converting enzyme
HPLC: High-performance liquid chromatography
LC–MS: Liquid chromatography−mass spectrometry
ABTS^+^: 2,2′-Azino-bis(3-ethylbenzothiazoline-6-sulfonic acid)
TPC: Total phenolic content
